# Tracing low-level structures in cryo-electron tomography

**DOI:** 10.1371/journal.pone.0338502

**Published:** 2025-12-12

**Authors:** Pelayo Alvarez Brecht, Francisco Aguilar-Martínez, Christian Biertümpfel, Irene Diaz, Naoko Mizuno, Antonio Martinez-Sanchez

**Affiliations:** 1 NIH Graduate Partnership Program, Bethesda, Maryland, United States of America; 2 Graduate student at the University of Oviedo, Department Computer Sciences, Campus Llamaquique, Oviedo, Spain; 3 Laboratory of Structural Cell Biology, National Heart, Lung, and Blood Institute, National Institutes of Health, Bethesda, Maryland, United States of America; 4 Department of Information and Communications Engineering, Universidad de Murcia, Murcia, Spain; 5 University of Oviedo, Department Computer Sciences, Oviedo, Spain; Tsinghua University, CHINA

## Abstract

Cryo-electron tomography is an imaging technique that provides 3D images (tomograms) *in situ* of cells with sub-nanometer resolution. Typically, the first step in the analysis is to classify the tomogram voxels into different structures, named semantic segmentation. However, the segmentation results are sets of voxels, hindering further quantitative analysis. In this paper, we define and implement algorithms to convert the semantic segmentation of the main structures in a cellular cryo-electron tomogram (membranes, filaments, cytosolic and membrane-bound macromolecules) into specific skeletons, preserving their topological and geometrical information. Additionally, we have defined a metric for comparing segmentations in cryo-ET coming from different methods more robust than the standard DICE. We also demonstrate how this approach can be used to trace cellular features by analyzing several *in situ* cellular cryo-electron tomograms.

## 1 Introduction

Cryo-electron tomography (cryo-ET) is an imaging technique that reconstructs three-dimensional (3D) images of intact cells at sub-nanometer resolution. These reconstructed volumes are also known as cryo-tomograms, or just tomograms. To facilitate quantitative analysis of cellular phenomena, the image processing workflow has been actively developed [1], including segmentation, annotation, statistical analysis, and sub-tomogram averaging.

The first step of the analysis workflow of a tomogram is typically semantic segmentation. The segmentation task classifies image pixels, or voxels in the case of tomograms, according to their corresponding structure (type of object). If all voxels have been associated with a class, including background, it is called semantic segmentation. For some years, there have been widely used methods to assist the segmentation of specific structures in cryo-ET, like macromolecules [2], filaments [3], or membranes [4]. These methods typically involve the pre-computation of a correlation or a saliency map. This step enhances a specific structure of interest. Afterward, a post-processing step generates the final segmentation. Recently, convolutional neural networks (CNN) [5] have shown great success in computer vision, and more specifically, U-Net [6] emerged as the most widely used neural network for biomedical image segmentation. Deep learning approaches are being adapted for cryo-ET, initially by segmenting generic structures in 2D slices [7], more recent methods try to integrate 2D and 3D information [8], or specialize in specific structures such as macromolecules [9], filaments [10] and membranes [11].

A raw segmentation does not represent the topology of the structures. Therefore, before performing a quantitative analysis, we must convert the segmentation into other data representations, retaining geometrical and topological properties. Moreover, topological information is necessary to compare accurately the segmentations obtained using different methods. As an example, skeleton centerlines must be extracted from filament segmentation to obtain quantitative information, as these 3D curves encode geometrical properties such as length, orientation, or curvature [12,13]. Additionally, it is necessary to obtain a graph representing interconnections to analyze the topology of filament networks. Although there are specific solutions for extracting centerlines of major cellular cytoskeleton structures like actin filaments or microtubules [3,10], there is no generic solution to trace filament-like structures, including networks with arbitrary topology. Similarly, segmentations made manually or by different algorithms tend to have a non-uniform thickness [14] along membranes, even within a single tomogram. As a consequence, it is hard to compare membrane segmentations of the same tomogram obtained by different methods. Regarding macromolecules, deep learning algorithms for segmentation do not provide center localizations directly; these must be approximated afterward [15].

In this paper, we present new methods to construct a processing workflow for efficiently extracting representations of the topology and geometry of the three main types of structures present in cryo-ET: membranes, filaments, and particles (or macromolecular complexes, either cytosolic or membrane bound). We define these objects as follows.

- Membranes: Surface-like structures that encompass the cells and organelles.
- Filaments: Line-like or net-like structures typically correspond to cytoskeleton elements such as microtubules or actin filaments.
- Particles: Blob-like structures that are identified with some macromolecules, some of them floating in the cell cytoplasm or the lumen of some organelles, and others bound to membranes.

Finally, we also demonstrate the versatile use of our workflow, which enables quantitative analysis by processing major cellular structures of several *in situ* cryo-electron tomograms.

## 2 Methods developed

**Fig 1:**
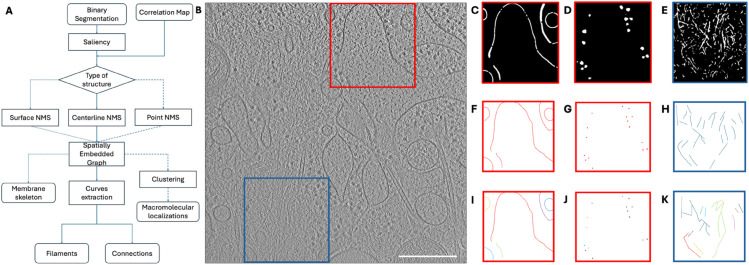
The workflow. (A) A scheme of the workflow; solid arrows depict the common steps and the path for filaments, dotted arrows the path for membranes, and dashed ones for macromolecules. (B) A 2D slice of the tomogram provided by [16] authors. Scale bar 200 nm. View of the red box with segmentation for membranes (C) and macromolecules (D). View of the blue box with filaments segmentation (E). Segmentations in (C-E) are not homogeneous, e.g. membranes do not have a constant thickness. (F-H) The results of applying NMS on (C-E) respectively. (I) Instance segmentation from the membranes traced in (F). (J) Positions of the macromolecules obtained from (G). (K) Curves extracted from the graph in (H).

This section describes the methods developed for the workflow summarized in Fig 1. The input is a semantic segmentation. Second, the segmented voxels of the structure of interest are isolated in a binary segmentation that is converted into a saliency map afterward. Alternatively, if available, the saliency can be provided as input directly. For filaments, the skeletons obtained from the saliency maps can be represented by a spatially embedded graph representation that unifies geometrical and topological information. The method used for generating the skeletons is based on Non-Maximum Suppression (NMS), but its application is different depending on the type of structure. In the case of filaments, we can further process the spatially embedded graph due to their specific topology, where each filament is modeled as 3D curves with their associated geometry. In the case of macromolecules, we can provide their localization by applying clustering methods.

The workflow is modular, and this work develops the methods for postprocessing the segmentation. Any existing method for semantic segmentation can be used to provide the input, including manual segmentation.

### 2.1 Saliency

A saliency map in tomography is a scalar field where voxel values indicate the likelihood of belonging to a specific structure. Formally, we can define the saliency map as $S:{\mathbb{R}}^{3}⟶\mathbb{R}$, twice continuously differentiable $S\in {𝒞}^{2}$, and positive-definite. In this context, each voxel, *i*, is associated with a vector of coordinates $x_{i}=(x_{i},y_{i},z_{i})\in {\mathbb{R}}^{3}$.

For instance, the probability or regression maps provided by some neural networks can be considered saliency maps. However, the output provided during the analysis, whether manual or software-based, often takes the form of segmentation. Still, the next step in the workflow, NMS, requires a differentiable scalar map as input. Therefore, this step is only required when the input is a binary segmentation, and its purpose is to generate a smooth saliency map.

**Fig 2:**
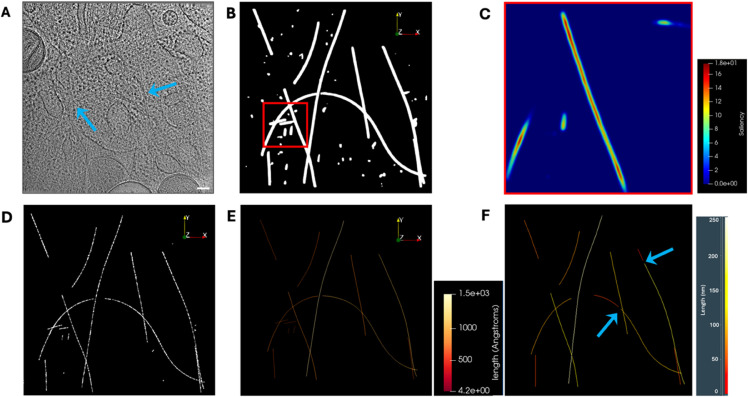
The saliency and NMS. (A) A 2D slice of a tomogram region with MTs approximately parallel to the X-Y plane, marked by the blue arrows. Scale bar 100 nm. (B) Microtubule segmentation in 3D. (C) Saliency map obtained from the segmentation. This is a 2D slice of the red box marked in B. (D) NMS. (E) NMS with the 3D curves extracted, the color encodes the curve length. (F) Filaments extracted with Amira [3], blue arrows point to fractures artificially created by Amira but not present in our procedure E, each color represents an instance. Colormaps in panels (E) and (F) are different because Paraview and Amira have different ones. Also, Paraview shows distance in Angstroms and Amira in nm.

When a structure is segmented, especially when it is manually done, the definition of its border can be arbitrary, resulting in inconsistent thickness across different instances. Nevertheless, we can assume that the voxels defining the skeleton of a segmented region are likely to belong to the structure. To capture this, we apply the distance transform [17] to the input segmentation, where values at the foreground encode the Euclidean distance to the nearest background voxel. Despite this, the scalar map is not yet the differentiable scalar field required by NMS. Therefore, we apply a Gaussian filter to produce a smooth and differentiable scalar map. The ridges of this scalar map correspond to the skeleton of the segmentation. See Fig 2A–2C.

### 2.2 Non-maximum suppression

The purpose of this step is to obtain the 3D skeleton of the structures targeted in a saliency map. The algorithm for selecting voxels belonging to local maxima is named non-maxima-suppression (NMS). Therefore, its output is a binary segmented tomogram. Here, we implement NMS by finding local maxima along the directions defined by the eigenvectors of the Hessian tensor (2nd derivative). The eigenvalues and eigenvectors of the tensor are required to determine the dimension of the local maxima. The tensor structure (1st derivative) is not used for obtaining directions since it is zero at local maxima.

The Hessian tensor of *S* is defined as

$$H_{S}=[\begin{array}{ccc} S_{xx} & S_{xy} & S_{xz}\\ S_{xy} & S_{yy} & S_{yz}\\ S_{xz} & S_{yz} & S_{zz} \end{array}],$$ (1)

where *S*_*i*,*j*_ are the second order partial derivatives $\frac{\partial^{2}S}{\partial i\partial j}$ with i,j∈{x,y,z}. We compute the 1st derivatives by the direct subtraction of the saliency of adjacent voxels along the direction of the derivative. Afterward, the 2nd derivatives are obtained by computing 1st derivatives using as input the corresponding 1st derivative.

The Hessian tensor *H*_*S*_ can be computed for every point x in *S*. From $H_{S}(x)$ three eigenvalues $\lambda_{1},\lambda_{2},\lambda_{3}$ can be obtained. The fastest approach to solve the eigenproblem for 3×3 matrices was proposed in [18], which solves the third-grade equation by using the Cardano method, being faster than the conventional iterative algorithm. Solving the eigen-problem generates three eigenvalues $\lambda_{i}$ with i∈{1,2,3} considering $\left|\lambda_{1}|\geq |\lambda_{2}|\geq |\lambda_{3}\right|$, and their associated eigenvectors $⃗v_{1}$, $⃗v_{2}$, and $⃗v_{3}$.

**Fig 3:**
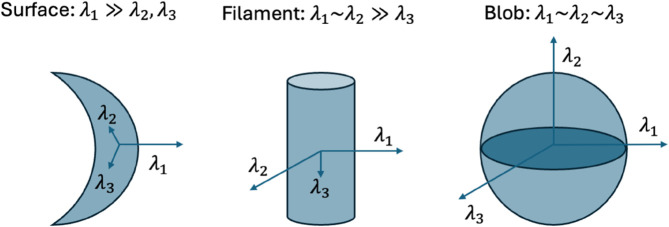
Dimension of the features. The analysis of the eigenvectors and eigenvalues determines the dimension of the features. Arrows represent the eigenvectors, and their length represents the eigenvalues.

The dimension, *d*, of a local maximum is characterized by the relation among eigenvalues as depicted in Fig 3. A 0-manifold (macromolecules) is associated with the case $\left|\lambda_{1}|\approx |\lambda_{2}|\approx |\lambda_{3}\right|$, 1-manifold (filaments) is $\left|\lambda_{1}|\approx |\lambda_{2}|>>|\lambda_{3}\right|$, and 2-manifold (membranes) is $\left|\lambda_{1}|>>|\lambda_{2}|\approx |\lambda_{3}\right|$. Consequently, at a blob center-point, $x_{i}\in {𝒮}_{0}$, *S* must have a local maximum simultaneously along the directions defined by $⃗v_{1}$, $⃗v_{2}$, and $⃗v_{3}$. Center-line points, $x_{i}\in {𝒮}_{1}$, in filaments only have a maximum along $⃗v_{1}$ and $⃗v_{2}$. Similarly, center-surface points, $x_{i}\in {𝒮}_{2}$, in membranes have a maximum just along $⃗v_{1}$. Here we used *>>* when the first element is at least one order of magnitude bigger than the second, so this one is depreciable, and *≈* when they have the same order of magnitude, and we have to take into account both.

So we can say that *S* can have three types of local maxima based on their dimension *d*: point with *d* = 0, line-like ridge with *d* = 1, and surface-like ridge with *d* = 2. The local maxima of dimension *d* are embedded on a *d*-manifold. Here, we define the skeleton of ${𝒮}_{d}$ at dimension *d* as the *d*-manifold containing all local maxima at such dimension. Therefore, we can use ${𝒮}_{0}$ for representing positions of macromolecules, ${𝒮}_{1}$ for filament center-lines, and ${𝒮}_{2}$ for membrane center-surfaces. Now, we can formally define NMS as ${𝒮}_{d}:{\mathbb{R}}^{3}\to \left\{0,1\right\}$, with *d* indexing the local maxima (or skeleton) dimension.

Algorithm 1 resumes how NMS is computed. The algorithm for computing NMS depends on the dimension for the skeleton, *d*. For a blob centered a point x, ${𝒮}_{0}$ skeleton, the three eigenvalues $\lambda_{1}$, $\lambda_{1}$ and $\lambda_{1}$ are comparable. Then we should investigate the saliency, *S*, values along the directions $⃗v_{1}$, $⃗v_{2}$, and $⃗v_{3}$ around point x, so it must present a local maximum simultaneously along these directions. To determine the variation of saliency at point $x_{i}$ along direction $⃗v_{j}$, we take $S_{i}^{j-}=S(x_{i}-\delta ⃗v_{j})$, $S_{i}=S(x_{i})$, and $S_{i}^{j+}=S(x_{i}+\delta ⃗v_{j})$, being *δ* a factor $\sqrt{3}$ of the voxel size. Typically, *S^j−^* and *S*^*j* + ^ do not fit exactly with a voxel center in *S*, so their value are computed by trilinear interpolation using information from the neighbor voxels (’trilinear’ function in Algorithm 1). Therefore, determining if $x_{i}$ is a local maxima along direction $⃗v_{j}$ consists of evaluating

$$(S_{i}>S_{i}^{j-})\wedge (S_{i}>S_{i}^{j+}).$$ (2)

For the case of filaments, ${𝒮}_{1}$ skeleton, it is only required to study the directions corresponding to larger eigenvalues, $⃗v_{1}$ and $⃗v_{2}$. Finally, for membranes, ${𝒮}_{2}$, only direction $⃗v_{1}$ is used. In Algorithm 1 the function ’sort_eigenvectors’ returns the eigen-vectors ordered according their corresponding eigen-values $\left|\lambda_{1}|\geq |\lambda_{2}|\geq |\lambda_{3}\right|$. In Eq 2, *S*^*j*−/ + ^ lies in between of eight voxels of the 3D saliency map, so their values are estimated by trilinear interpolation.

**Algorithm 1 Non-maximum suppression of a discrete scalar map.**

**Require:** *S* 3D scalar map. *L* vectorial map with the

eigenvalues. *V* vectorial map with the eigenvectors. *d*

skeleton dimension: "0" blobs, "1" filaments, "2"

membranes.

**Ensure:** *M* binary map.

1: M←zeros(shape(S))

2: **for** $x_{i}\in S$ **do**

3:   $\hat{d}\leftarrow 2-d+1$

4:   $\left(⃗v_{1},⃗v_{2},⃗v_{3})\leftarrow \text{sort\_eigenvectors}(L,V,i\right)$

5:   j←1

6:   **repeat**

7:    $S_{i}^{j-}\leftarrow \text{trilinear}(S(x_{i}-\delta ⃗v_{j}))$

8:    $S_{i}^{j+}\leftarrow \text{trilinear}(S(x_{i}+\delta ⃗v_{j}))$

9:    j←j+1

10:   **until** $(j\leq \hat{d})\wedge \left(S_{i}=\max([S_{i}^{j-},S_{i},S_{i}^{j+}])\right)$

11:   **if** $j>\hat{d}$ **then**

12:    $M(x_{i})\leftarrow 1$

13:   **end if**

14: **end for**

The final skeleton can also be seen as a point cloud. Consequently, it can be further subsampled to minimize the impact of false detections from NMS produced by numerical errors. Additionally, the subsampling alleviates the computational cost for the next steps without losing significant precision. For our example, we can see how it looks in Fig 2D.

### 2.3 Spatially embedded graph

A spatially embedded graph (SEG) is defined as a collection of points, called nodes or vertices, 𝒩, and a collection of pairs of nodes called edges ℰ:

$$𝒢=\{\{N_{1},N_{2},...,N_{n}\},\{(N_{i_{1}},N_{i_{2}}),...,(N_{i_{m_{1}}},N_{i_{m_{2}}})\}\}=\{𝒩,ℰ\}$$ (3)

For each node *N*_*i*_, we can associate the corresponding point $x\in {\mathbb{R}}^{3}$ containing the geometrical information. An edge $E_{i}=(N_{i_{j}},N_{i_{k}})$ determines how two nodes, $N_{i_{j}}$ and $N_{i_{k}}$ with $i_{j},i_{k}\in (1,...,n)$ and $i_{j}\neq i_{k}$, are interconnected, thus containing the topological information.

If the node i∈(1,...,n) is connected with the node j≠i, there is a path between *i* and *j*. A path is a group of edges that starts at node *N*_*i*_ and ends at *N*_*j*_. A cycle is a path that starts and finishes in the same node.

**Fig 4:**
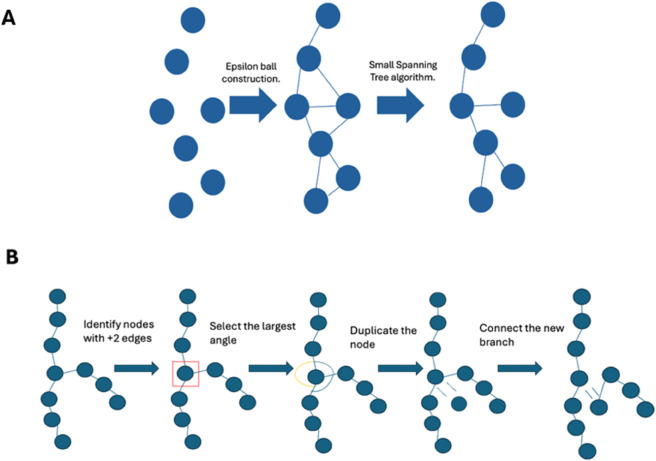
SEG processing. (A) Steps to construct the SEG from the NMS point cloud. Balls represent the nodes and lines the edges. (B) Steps to extract the filaments from the SEG. The duplicated node has the same coordinates as the original one (red box); we have shifted it for clarity.

Our aim is to obtain an acyclic SEG representation of ${𝒮}_{d}$ to represent its geometry and topology. An initial SEG, 𝒢, is constructed by creating a node for each voxel *i* in ${𝒮}_{d}$ and associating with this node the voxel’s coordinates $x_{i}$. Edges are created using the epsilon ball, *ε*, criterion. For each $x_{i}\in 𝒩$, if there is a $x_{j}$, i≠j, with $\|x_{i}-x_{j}\|<\epsilon$, then a new edge $\left(N_{i},N_{j}\right)$ is added to *E*. This process may generate cycles, but we remove the cycles using a minimum spanning tree algorithm [19]. This algorithm finds the smallest subgraph, ${𝒢}^{\prime }$, that connects all the nodes of the graph without cycles, determining the new acyclic graph. This algorithm may not generate unique graphs, but we are in a Euclidean space, where every edge is weighted by its length, then we prioritize the minimum length to get a unique graph. The only exception is the case of two paths having the same length, so any election is equally good for our purposes. These steps are summarized in the scheme of Fig 4A.

### 2.4 Model for filaments

On the one hand, the skeleton for macromolecules (blobs), *S*_0_, has a trivial topology as points are not interconnected. On the other hand, recovering the topology of surfaces of *S*_2_ is generally cumbersome. Here, we focus on constructing a data structure that unifies topological and geometrical information specifically for the skeletons of filamentous structures, *S*_1_. For this purpose, we use the spatially embedded graphs described above.

Cellular filamentous polymers typically form independent chains, e.g. microtubules, or in some cases, like actin filaments, which may be interconnected through molecular complexes, resulting in tree-like networks. Consequently, the next processing step for the SEG is to instantiate the different filaments within the acyclic graph. A filament is a subgraph, ${𝒢}^{f}=({𝒩}^{f},{ℰ}^{f})\subseteq 𝒢$, where nodes are ${𝒩}^{f}=(N_{1},N_{2},...,N_{n})$ and edges ${ℰ}^{f}=\left((E_{1},E_{2}),(E_{2},E_{3}),...,(E_{n-1},E_{n})\right)$. Algorithm 2 defines the procedure to obtain the filaments that compound an acyclic graph. This algorithm first identifies nodes that correspond with filament branches, those with more than 2 edges. Second, for each of these nodes, *N*_*i*_, the shortest angles formed by all pairs of edges are computed. The pair of edges forming the largest angle is preserved while the rest are removed. Finally, for each removed edge, a new node is created in the same position as *N*_*i*_, then an edge is added to connect the new node with the non-*i* node of the deleted edge. The steps to construct a SEG with the filaments instantiated are summarized in Fig 4B.

**Algorithm 2 Filament instances from a SEG.**

**Require:** 𝒢; a spatially embedded graph.

**Ensure:** ${𝒢}^{\prime }$; graph with filaments disconnected

${𝒢}^{\prime }\leftarrow copy(𝒢)$.

$\left(N_{1},...,N_{n})\leftarrow nodes({𝒢}^{\prime }\right)$

**for** i←1 **to** *n* **do**

$ℰ=(E_{1},...,E_{m})\leftarrow edges(N_{i})$ {The *m* edges of node *N*_*i*_}

**if** *m*>2 **then**

$\alpha_{max}=0$

𝒫←∅

**for** j←1 **to** *m* **do**

$(N_{i},N_{p})\leftarrow E_{j}$

**for** k←j+1 **to** *m* **do**

$(N_{i},N_{q})\leftarrow E_{k}$

$\alpha =angle(N_{p},N_{i},N_{q})$

**if** $\alpha >\alpha_{max}$ **then**

$P\leftarrow (E_{j},E_{k})$

$\alpha_{max}=\alpha$

**end if**

**end for**

**end for**

${ℰ}^{\prime }\leftarrow remove\_edges(P,ℰ)$ {${ℰ}^{\prime }$ contains the edges to disconnect}

$l\leftarrow number\_nodes({𝒢}^{\prime })$

**for** j←1 **to** *m*–2 **do**

l←l+1

$add\_node(N_{l},{𝒢}^{\prime })$

$set\_position(N_{l},x_{i})$

$\left(N_{i},N_{k})\leftarrow remove\_edge({ℰ}^{\prime }(j),{𝒢}^{\prime }\right)$

$add\_edge((N_{l},N_{k}),{𝒢}^{\prime })$

**end for**

**end if**

**end for**

In our case, graphs rarely contain nodes with more than three adjacent nodes, so the computational complexity of Algorithm 2 is *O*(*n*), being *n* the number of nodes. The output of Algorithm 2 is another SEG, ${𝒢}^{\prime }$, containing connected nodes for every filament and disconnected to the nodes of any other, as well as ensuring that every filament is a curve-like sub-graph $ℱ=((N_{1},N_{2},...,N_{n_{f}}),((E_{1},E_{2}),(E_{2},E_{3}),...,(E_{n_{f-1}},E_{n_{f}}))$. Consequently, every filament ℱ represents a curve in ${\mathbb{R}}^{3}$ space defined by the discrete point sequence $C_{F}=(x_{1},x_{2},...,x_{n_{f}})$. The geometrical parameters, local and global, of the discrete curves are numerically computed as described in [20]. Local parameters vary at every point along the curve, while global parameters have a single value characterizing the entire curve, see Fig 2E–2F. Local parameters include geodesic distance to origin, tangent vector, the signed and unsigned curvatures, and torsions. Global parameters include geodesic length, sinuosity, persistence length, and total curvature and torsion.

### 2.5 Mean shift clustering

The skeleton ${𝒮}_{0}$ represents the localization of macromolecules. The input segmentation, or saliency maps, contains blobs that may generate more than one maxima per macromolecule. Consequently, it is necessary to cluster the point cloud of ${𝒮}_{0}$, generated by NMS, to obtain a single point per macromolecule. Similarly to [21], we apply the Mean Shift [22] algorithm by adjusting the bandwidth to macromolecule diameter. This approach performs well if all macromolecules in a segmentation have approximately the same radius.

### 2.6 Revising DICE metric

Another potential application of the skeletons generated in Sect 2.2 is to construct metrics that better evaluate segmentation accuracy in cryo-ET. Since the thickness of a feature may vary according to the segmentation approach used and even within the same segmentation, which is unavoidable for the case of manual segmentations, skeletons offer the advantage of having a uniform thickness (approximately one voxel). Based on skeletons, Ref. [23] proposes clDice (centerline Dice) for tube-like structures in 2D images. This metric has recently been extended to 3D structures [11,24]. The advantages of this metric are: (1) its versatility as two segmentations of the same structures can be compared regardless of how the segmentation was generated, (2) its computation is fast because the skeleton computation is CPU parallelized (see Sect 3).

As proposed in [23], topological DICE is computed from the topological precision *TP* and the topological sensitivity *TS*. These values are computed by analyzing the overlap between two segmentations. Besides the input *S^in^* –the segmentation to evaluate– and the reference *S^ref^* –the ground truth–, here we introduce the notion of dimension, *d*, for the respective skeletons, ${𝒮}_{d}^{in}$ and ${𝒮}_{d}^{ref}$, then the computation of these values becomes:

$$\begin{array}{cc} TP_{d}=\frac{\left|S_{d}^{in}\cap {𝒮}^{ref}\right|}{\left|S_{d}^{in}\right|} & TS_{d}=\frac{\left|S_{d}^{ref}\cap {𝒮}^{in}\right|}{\left|S_{d}^{ref}\right|} \end{array}$$ (4)

Finally, the revised *DICE*_*d*_ at dimension *d* is the harmonic mean of these values

$$DICE_{d}=\frac{2\cdot TP_{d}\cdot TS_{d}}{TP_{d}+TS_{d}}$$ (5)

Although our revised *DICE*_*d*_ metrics follow the same approach proposed in [23], they have been extended to, first, process 3D images instead of 2D, and second, process three different features (blobs, lines in 3D, and surfaces) instead of just lines in 2D. The revised *DICE*_*d*_ is not just the standard DICE, but with skeletons. The differences between the above equations compared to standard DICE consist of two details. Firstly, the dimension *d* of the feature, which determines how to compute the skeleton as explained in Sect 2.2. Secondly, the revised *DICE*_*d*_ requires both full segmentations, *S^in^* and *S^ref^*, and their topological information, that is, their skeletons, $S_{d}^{in}$ and $S_{d}^{ref}$, to measure the *TP*_*d*_ as the fraction of $S_{d}^{in}$ within *S^ref^*, and *TS*_*d*_ as the fraction of $S_{d}^{ref}$ in *S^in^*.

### 2.7 The datasets

The data used for validating the methods developed, see Sect 4, has been collected from three different sources. First, the public dataset in CryoET Data Portal under ID DS-10445, which was deposited for the competition for particle detection in cryo-ET [25]. This dataset contains 120 experimental tomograms with a reliable ground truth for macromolecules and membrane segmentation by Membrain-Seg [11], which were collected with a tilting range of $-45.03^{\circ }$ to $44.97^{\circ }$ and a voxel size of 1.0012 nm. Second, we generated a synthetic dataset with PolNet [24], which has been deposited in Zenodo with DOI 10.5281/zenodo.16528986. This dataset contains 120 synthetic tomograms with a reliable ground truth for actin-like filaments, which were collected with a tilting scheme of $-60^{\circ }$ to $60^{\circ }$ and a voxel size of 1 nm. Third, three *in situ* tomograms collected by the authors and collaborators, deposited also in Zenodo with DOI 10.5281/zenodo.16528986. The *in situ* tomograms contain all features but no ground truth with voxel sizes of 1.062 nm and 1.648 nm and a tilting range of $-60^{\circ }$ to $60^{\circ }$. These datasets were deposited and processed under the terms and conditions of their respective databases.

## 3 Implementations details

All these algorithms are integrated into the package TracET. We release TracET as a Python package to make it compatible with most cryo-ET software. On the one hand, end users can have direct access to the algorithms by running the specifically prepared Python scripts. On the other hand, software developers can include TracET package in their Python developments. Available at https://github.com/PelayoAlvarezBrecht/tracET.git

Some routines are computationally intensive, specifically solving the eigenproblem and the NMS. The eigenproblem has been implemented using the approach described in [18] for 3x3 matrices, and both algorithms have been CPU parallelized. Consequently, the code for these algorithms has been written in C to alleviate the running times. We utilized NumPy C-API to abstract users from the C code details, thus providing a Python interface for TracET functionality. We have used NetworkX library [26] for graph management, and Open3D [27] for point cloud processing.

## 4 Results

### 4.1 Quantitative validation

To validate TracET for postprocessing segmentations of macromolecules, we evaluate quantitatively TracET output with the public dataset CZII - CryoET Object Identification Challenge - Public Test Dataset with ID DS-10445 [25] described in Sect 2.7.

The purpose of this validation is to verify that TracET, for any dimension, extracts the skeletons correctly, retaining geometrical and topological information from an input segmentation. PolNet provides a segmentation ground truth for the filaments. In the CZII dataset, membranes are segmented using Membrain-seg. For the macromolecules, we constructed a segmentation for each type of particle using the coordinates provided by the CZII dataset and placing instances of the segmented particles with random orientations.

We define the next measures for determining TracET performance for macromolecules:

- TP (True positives): Number of center points correctly found.
- FP (False positives): center points found but not correctly assigned.
- FN (False negatives): coordinates in the ground truth without a center point assigned.
- F1-score: $F_{1}=\frac{2TP}{2TP+FP+FN}$.

*F*_1_ close to one means a good performance, so tracET is predicting accurately most of the particle positions. A center point for a macromolecule found is correctly assigned if it is closer than half of the particle radius to a ground truth coordinate.

Table 1 contains the results for the macromolecules. The dataset has 5 different types of particles with radii specified in the table. The metrics are for the total number of particles in all tomograms. We also add the standard deviation of the *F*_1_ measure for the total of particles in every single tomogram. The results validate the accuracy (high *F*_1_) and robustness of TracET (low $\sigma_{F_{1}}$) in determining the center of different macromolecules from blob segmentations.

**Table 1:** Validation for blob center detection.

Particle	Radius (Å)	TP	FP	FN	$F_{1}$	$\sigma_{F_{1}}$
**Apo-ferritin**	60	9105	1391	43	0.927	0.0104
**Beta-galactosidase**	90	3695	67	33	0.987	0.0180
**Ribosome**	150	10825	109	34	0.993	0.00732
**Thyroglobulin**	130	5372	1979	8	0.844	0.00948
**Virus-like-particle**	135	1060	17	0	0.992	0.00147
**Citosolyc particles (Total)**	-	30057	3563	118	0.942	0.0134

For membranes, we use the same dataset as for macromolecules. Now, we verify that the center surface, skeleton of *d* = 2, generated by TracET, is inside the membrane segmentation provided in the dataset.

- Correct points. Points of the *d* = 2 skeleton that are inside the segmentation.
- Failed points. Points of the skeleton that are outside of the segmentation.
-
$Precision=\frac{Failed\_points}{Correct\_points}$

The first row of Table 2 shows that almost all points of the skeleton *d* = 2 generated by TracET are inside the original membrane segmentation for all tomograms. These results validate that TracET does not introduce errors during the postprocessing. Additionally, considering that *d* = 2 is one voxel thick and completely covers the membrane, they also confirm that the skeleton can be used to represent membrane geometry and topology.

**Table 2:** Validation for membranes, d=2, and filaments, d=1, skeletons.

Dimension	Correct Points	Failed points	Precision	$\sigma_{Precision}$
*d* = 2	838346	6783	0.992	0.0193
*d* = 1	4044013	203098	0.952	0.00622

Finally, for filaments, we use synthetic data generated by PolNet described in Sect 2.7. The measures to evaluate the performance of TracET in this case are the same as those we use for membranes. The results for filaments are in Table 2 second row. Here we have a larger number of points, because these tomograms are designed to have lots of filaments. Anyway, we can see that the precision of TracET is also high and consistent among all the measurements.

### 4.2 Application examples

In this section, we present examples for processing experimental cryo-electron tomograms to demonstrate potential applications of the methods developed and described in Sects 2 and 3.

To provide an input to TracET, we construct a UNet network for the semantic segmentation of 3D images using nn-UNet [28]. This network was trained using 10 synthetic tomograms generated by PolNet software. However, the label field generated by PolNet was substituted by a modified version. For example, in the case of microtubules, PolNet generates a ground truth segmenting the shell of microtubules but not their centerline. Consequently, we have developed a procedure to generate an appropriate label field from the PolNet motif list, which includes center-surface for membranes, center-line for filaments (including microtubules), and center-points for macromolecules. Specifically, all local features have been approximated to spheres with different radii: 2.5 nm for membranes, 6 nm for microtubules, 4 nm for actin filaments, 6 nm for ribosomes, 2.5 nm for other cytosolic macromolecules, and 5 nm membrane bound proteins.

**Fig 5:**
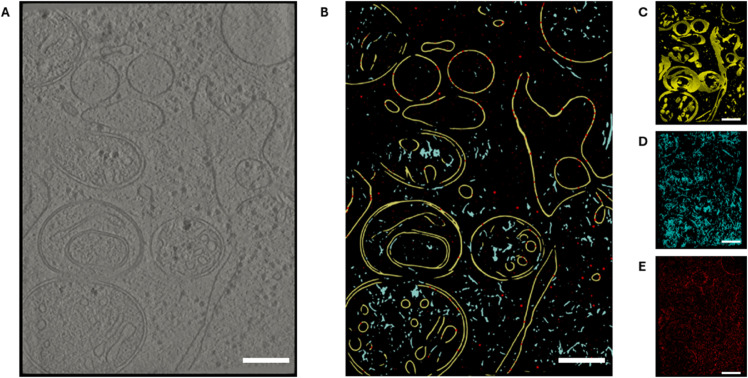
Segmentation process. (A) A 2D slice of a cellular tomogram. (B) The segmented tomogram, with all the elements together but having different labels. The elements separated: membranes (C), filaments (D), and macromolecules (E). The color code for all panels is: Yellow for membranes, blue for filaments, and red for all types of macromolecules. Scale bars 200 nm.

Therefore, a model trained with the modified ground truth performs semantic segmentations with 7 classes: 0 background, 1 membranes, 2 microtubules, 3 filaments, 4 ribosomes, 5 other cytosolic macromolecules, and 6 membrane bound macromolecules. Membranes are segmented as a single sheet with a thickness approximated to 5 nm (lipid bilayer), microtubules as solid cylinders with 6 nm of axial radius, filaments as solid cylinders with 4 nm radius, and macromolecules as spheres with the radii specified before. In Fig 5, we show the result of this segmentation with an exemplary *in situ* tomogram.

Data was processed using a workstation running Ubuntu 20.04.6 LTS with an Intel Xeon Gold 6148 2.40GHz processor, 512GB RAM, and a NVIDIA RTX A5000 GPU and 6 NVIDIA GeForce RTX 2080.

#### 4.2.1 Membrane 3D skeleton.

We have taken two *in situ* cryo-electron tomograms with membranes from large vesicles, the endoplasmic reticulum, and mitochondria. These tomograms have dimensions of T1: 1023×1440×100 and T2: 1023×1440×135 voxels, with a voxel size of 1.062 nm.

**Fig 6:**
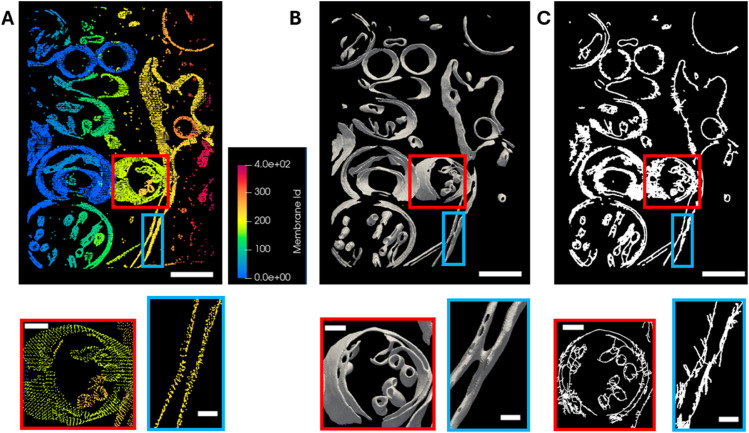
Membrane segmentation. (A) Membrane analysis with TracET workflow. Membranes have been classified by the application of a connectivity analysis, and the color code corresponds with the label of every membrane. (B) Membrane segmentation with Membrain. (C) 2D Skeleton from the Membrain segmentation obtained by Skeletonize3D. Scale bars are 200 nm. All panels have zooms to the boxed regions below. Scale bars for zooms are 20 nm. The original tomogram slice can be seen in Fig 5A.

These tomograms were processed using two different methods, Membrain-Seg and the model presented in Sect 4.2, which obtained Fig 5C membrane segmentation, to provide an input to TracET. Fig 6A–6B shows the side-by-side membrane segmentation. Both methods provide a similar overall segmentation, but the thickness of the segmented membranes is different for each method. In the case of TracET workflow, we have obtained a subsampled version of the *S*_2_ skeleton. Therefore, the segmentation is less dense but contains similar structural information. For comparison, we have also computed the skeleton using the Skeletonize tool, see Fig 6C. Skeletonize uses LeeÂ´s method [29], being available at Scikit-Image Python package [30]. In contrast to TracET, the Skeletonize tool fails to retain the 3D topological information.

Table 3 shows the revised *DICE*_2_ measures and intermediates, see Sect 2.6, using TracET output as the input segmentation and MemBrain segmentation as the reference. Because the values are high, we can consider that the segmentation model presented in Sect 4.2 approximates the behavior of Membrain for the tomograms processed. The Sect 4.4 contains a study validating the higher robustness of the revised *DICE*_*d*_ than the standard DICE for comparing segmentations.

**Table 3:** Revised, or topological, $DICE_{2}$ comparing membrane segmentations of TracET and MemBrain.

	$TP_{2}$	$TS_{2}$	$DICE_{2}$
**Tomogram 1**	0.811	0.749	0.779
**Tomogram 2**	0.752	0.626	0.683

#### 4.2.2 Tracing filament networks.

Here, we apply TracET to analyze filamentous structures in cellular tomograms. Currently, the most used tool is the filament tracer of Amira [3]. Here, we process an *in situ* tomogram, with dimensions T3: 926×926×250 voxels and a voxel size of 1.648 nm, provided by [16] authors, which contains many other cellular features besides microtubules. Amira tracing tool begins by computing the maximum cross-correlation map of the tomogram against a microtubule template over the entire space of rotations, a step analogous to our saliency map generation. The microtubule template was generated using the following parameters: cylinder length 80 nm, outer radius 12.1 nm, inner radius 10 nm, and mask radius of 15 nm. The rotation space was sampled with 5 degree angular increment. Next, we use Amira’s tool to trace the correlation lines with the following parameters: minimum seed correlation 68, minimum continuation quality 45, direction coefficient 0.3, minimum distance 24.2 nm, minimum length 80 nm, and search cone with a length 80 nm, 5 degrees angle, and a minimum step size 10%. Conversely, our workflow requires far fewer free parameters, just three: Gaussian saliency filter σ=2 voxels, saliency threshold 0.3 for NMS, subsampling of 1 every 10 voxels (every 6.06 nm), and radius for graph construction, *ε*, of 15 voxels (9.10 nm). Furthermore, despite an extensive optimization of the Amira parameters, our approach still outperforms Amira. TracET traces single instances of highly curved microtubules; meanwhile, Amira fractions them in some instances, as shown in Fig 2E–2F. Also, TracET is faster than Amira, as we can see in Sect 4.3.

**Fig 7:**
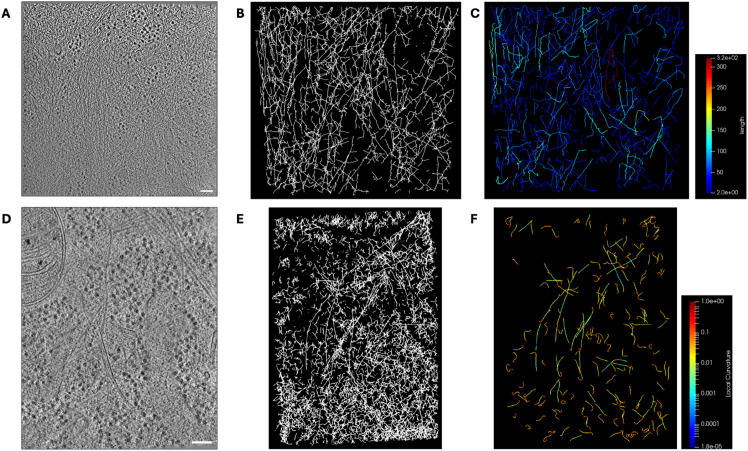
Tracing filaments. (A) and (D) 2D slices of *in situ* tomograms with a dense network of filaments. (B) and (E) the SEG obtained from tomograms A and D respectively. (C) and (F) curves extracted from B and E respectively, filtered by length with a threshold of 85 nm. In C the color encodes the length of the curves, a global property, and in F the local curvature, a local property.

Our workflow is versatile and can be used to trace filamentous structures with any topology. For example, we successfully traced filamentous structures in *in situ* tomograms, see Fig 7. The SEG resumes the geometry and topology of the filament network independently of its complexity. In addition, the geometrical properties, local and global, of the curves representing the filaments are computed. A complete list of these properties is available at Sect 2.4.

#### 4.2.3 Localizing macromolecules.

**Fig 8:**
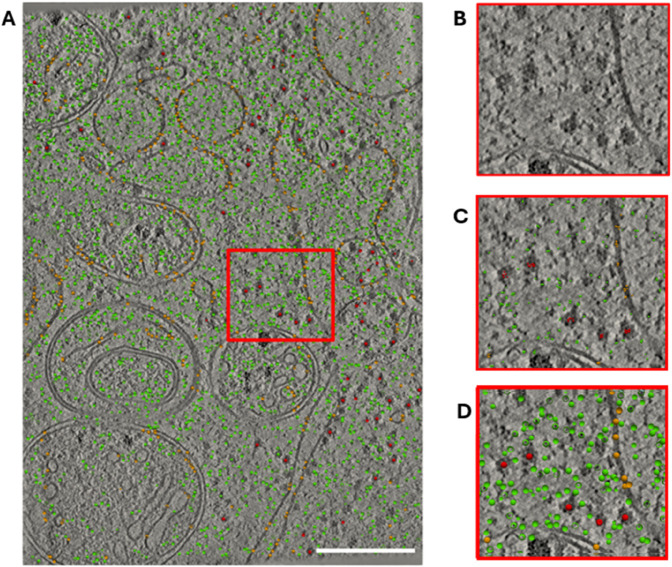
Detection of the macromolecular centers. (A) 2D slice of an *in situ* tomogram with the segmented ribosomes (red), cytosolic particles (green), and membrane-bound macromolecules (orange) segmented. (B) A zoom of the tomogram with the background context of the particles selected, a cluster of ribosomes is visible. (C) The skeleton with the local minima of the macromolecules in B. (D) The spheres represent the particles in C finally detected by using MeanShift. The radius of the spheres. Scale bar 200 nm.

The segmentation method used is capable of segmenting three different groups of macromolecules: ribosomes, other cytoplasmic complexes, and membrane-bound complexes. TracET returns the center points of these segmentations by adjusting the meanshift bandwidth parameter to the sphere radius used for training the neural network, that is, 6 nm for ribosomes, 2.5 nm for other cytosolic macromolecules, and 5 nm for membrane-bound proteins. Fig 8 shows the results of applying the workflow on a *in situ* tomogram to analyze the distributions of macromolecules. As expected, ribosomes present a clustered organization due to polyribosomes, and we verify that membrane-bound proteins colocalize with membranes.

### 4.3 Running times

The running times for processing the experimental tomograms used in Figs 2 and 7 are summarized in Table 4. In comparison with the Amira template matching, based on computing thousands of cross-correlations, our segmentation and saliency steps are faster. Additionally, the running times for tracing steps, NMS, SEG, and Curves, are lower than their analogous in Amira. Table 5 contains the times of Amira. It is important to notice that Amira could only process microtubules; therefore, time comparisons are done only for tomogram T3.

**Table 4:** Running times in seconds of every step in the workflow.

	T.	Seg.	Sal.	NMS	SEG	Curv.	Clust.	TOTAL
**Membranes**	T1	148	129	68	-	-	9	354
	T2	152	164	84	-	-	7	407
**Microtubules**	T3	905	921	398	52	52	-	2358
**Actin F**.	T2	152	221	103	2321	3631	-	6428
	T3	905	724	397	1030	2974	-	6000
**Ribosomes**	T1	148	167	90	-	-	21	426
	T2	152	139	65	-	-	12	368
**Cyto. Prot**.	T1	148	189	121	-	-	193	651
	T2	152	124	66	-	-	131	473
**Memb. Prot**	T1	148	172	92	-	-	28	440
	T2	152	125	65	-	-	29	371

**Table 5:** Running times in seconds for Amira, only used for microtubules.

	T. Size	Temp. Match.	Trac. Lin.	TOTAL
**Microtubules**	T3	2983	712	3695

### 4.4 Comparing segmentations and DICE behavior

**Fig 9:**
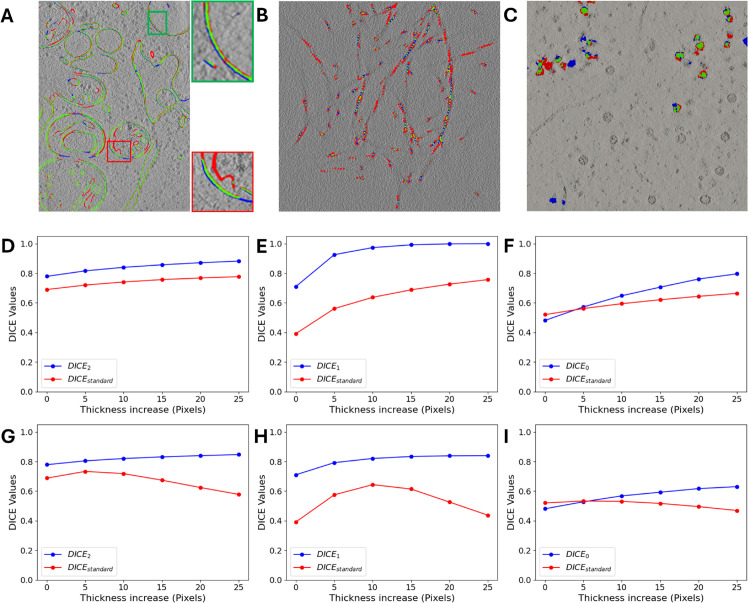
Robustness of the revised *DICE*_*d*_. (A-C) shows an input segmentation in blue, a segmentation used as ground truth in red, and the intersection in green, for dimension *d* = 2 (membranes), A, *d* = 1 (filaments), B, and *d* = 0 (blobs), C. Panel A has zooms to see that displacements and differences in the thickness of the segmentation contribute to reduce the value of the standard DICE. (D-F) compare the standard DICE and revised *DICE*_*d*_ for dimensions *d* = 2, *d* = 1, *d* = 0, increasing the thickness of both input and reference segmentations. (G-I) compare the same metrics but only by increasing the thickness of the input segmentation.

In this section, we study the behavior of our revised *DICE*_*d*_ to show that it is a more suitable option to compare different segmentations than the standard one for cryo-ET. One of the main problems of the standard DICE to compare two segmentations is that it requires the input and ground truth to have similar thickness. Therefore, standard DICE does not work properly to compare segmentations from different methods; these problems are evident in Fig 9A–9C. These figure panels contain a comparison of two segmentations for the three different dimensions. For instance, the panel Fig 9A compares two segmentations of membranes, *d* = 2, the segmentation to be evaluated in blue and the ground truth in red. Despite having a similar topology, the fraction of the overlapping region between both segmentations, in green, underestimates the actual agreement for representing the membrane shape. This effect is more evident in the zoomed green region, where both segmentations completely agree regarding the membranes center-surface, which resumes the membrane shape. However, many voxels remain in red o blue, thus penalizing the standard DICE, because of differences in the thickness between the segmentations. Our revised measures, by comparing the skeletons and segmentations, are independent of the thickness.

Fig 9A shows the comparison of two membrane segmentations made by two methods. The model proposed in Sect 4.2 and Membrain-Seg. Fig 9B shows the comparison of two filament segmentations. One is the ground truth provided by PolNet, segmenting accurately the position of every monomer of actin polymers, and the second is a line that simply defines the filaments’ centerline. Fig 9C shows the ribosome segmentation from two different methods. The segmentation generated by the model proposed in Sect 4.2 and another constructed from the ground truth coordinates provided by the CZII Dataset ID DS-10445. For each dimension, we use two different segmentation methods to compare two segmentations of the same structure on the same data.

Fig 9D–9I shows the overall higher robustness of our revised *DICE*_*d*_, in comparison to the standard one. We understand robustness as the resistance of the measures to variations in the segmentation’s thickness by dilation, which do not considerably modify the topology. In general, the segmentations compared retain similar geometrical and topological information, but little differences, like thickness, which does not affect the topology of the segmentation, reduce the value of the standard DICE. In addition, we can increase these differences by applying dilation to the segmentations. Fig 9D–9F shows that the revised *DICE*_*d*_ obtains higher values in all dimensions than the standard DICE when the dilation is the same in both segmentations. This is because in the revised *DICE*_*d*_, we compute the fraction of the skeletons within the full segmentations. Conversely, the standard DICE compares directly the segmentations, so disagreement in the thickness will penalize the measure.

Fig 9G–9I shows that under dilations for only one segmentation, the standard DICE in all dimensions drops when the thickness differences increase. Conversely, the revised *DICE*_*d*_ keeps a higher and more stable value because the skeletons are not affected by dilations.

## 5 Discussion

TracET relies on analyzing local geometrical and topological information to construct skeletons of the different features present in cryo-ET. The computation of the skeletons is a computationally intensive step. Here, we propose an efficient approach based on adapting NMS to the dimension of the skeleton. Classical approaches for computing 3D skeletons, with implementation in Fiji/ImageJ and Scikit-Image Python package [29–31] and MitoGraph [32], do not incorporate the concept of dimension into their skeletonization process, resulting in suboptimal results for surface-like structures such as membranes, or globular macromolecular structures. Furthermore, these implementations struggle to process tomograms with intricate networks such as filament networks present in cellular *in situ* cryo-electron tomograms. They encounter memory problems or fail to complete within a reasonable time frame, sometimes taking more than a day for cryo-electron tomograms. We tried to use Mitograph method to process the tomograms in Fig 7, but it did not converge. In Fig 6 we can see now a comparison of the output obtained with TracET, Fig 6A, and Skeletonize 3D [29], Fig 6C. The comparison clearly shows that TracET retains correctly the topological and geometrical information in 3D for membranes, but that is not the case for Skeletonize.

In conclusion, we present a versatile and efficient workflow to process the most common features in cryo-ET. For membranes, it traces the membrane surface-centerline with an approximate thickness of one pixel. For filaments, it constructs a spatially embedded graph that summarizes the geometry and topology of any arbitrary network, which also extracts its curves and computes its geometrical properties. Finally, the workflow detects macromolecules by determining the center of blob segmentations. Consequently, this workflow provides data structures to facilitate quantitative analyses from cryo-ET data. In addition, a revised *DICE*_*d*_ measure has been adapted to include the concept of dimension for robustly comparing segmentations in cryo-ET.
